# Editorial: Innovations in Functional Food Production: Application of Fruit and Vegetable Juices as Vehicles for Probiotic Delivery

**DOI:** 10.3389/fmicb.2022.854705

**Published:** 2022-03-07

**Authors:** Stavros Plessas

**Affiliations:** Laboratory of Food Processing, Department of Agricultural Development, Democritus University of Thrace, Orestiada, Greece

**Keywords:** fruit juices, probiotics, lactic acid bacteria, fermentation, health benefits

Nowadays, the innovations introduced in the food industry mainly focused on the production of functional food products (Misra et al., 2021). This trend is based on consumer awareness and demand for foods without chemical additives and with health benefits. Functional foods improve one or more target functions in the body, beyond their standard nutritional effects (Ashaolu, 2020). The major segments of functional food production by the Food Industry deal with fermented foods. In particular, probiotic foods are foods fermented through probiotic bacteria and are considered a major subcategory of functional foods. Probiotics are defined as live microorganisms that, when administered in adequate amounts, confer a health benefit on the host (Hill et al., 2014). The probiotic products in the market are mostly milk-based products, such as yogurts, cheese, and fermented milk. However, the use of milk-based probiotic products seems to be limited by allergies, cholesterol diseases, dyslipidemia, and veganism (Ranadheera et al., 2017). Therefore, wide research aiming at the production of innovative, alternative mediums for probiotic delivery has lately been seen.

Fruit and vegetable juices are considered a good alternative, especially taking into account that their market is one of the faster growing worldwide (Lillo-Pérez et al., 2021). Fermented fruits and vegetables are usually referred to as healthy foods, targeted at young and old people. They are vital foods of choice, due to their excellent functional and nutritional properties aided by fermenting probiotic microorganisms. The health benefits of fermented fruit and vegetable juices are mainly attributed to the presence of novel bioactive compounds in the form of phytochemicals, such as phenolics and fatty acids, as well as minerals and vitamins in considerable and higher amounts compared to their unfermented forms.

In this vein, this Research Topic highlights recent advances in the assessment of fruit and vegetable juices as “vehicles” for probiotic delivery. In addition, current knowledge regarding the amelioration of probiotic viability during fruit juices fermentation is addressed, as well as the production of novel functional bioactive compounds through fermentation of vegetable by-products.

The development of new functional fermented plant-based products is a very interesting perspective. In particular, soymilk is a plant product that seems an adequate medium for the growth of various beneficial bacteria such as bifidobacteria and various LAB. However, soymilk has not been examined as a substrate for the growth of probiotic propionibacteria. Likewise, the suitability of soymilk as a substrate for growth and acidification by *Propionibacterium freudenreichii* CIRM-BIA129 is discussed by Tarnaud et al. *P. freudenreichii* accomplished growth in soymilk in the presence of *Lactiplantibacillus plantarum*. Acid and bile tolerance of *P. freudenreichii* was enhanced in soymilk ultrafiltrate compared to milk ultrafiltrate. In addition, the culture medium modulated the number of stress proteins involved in stress remediation. Likewise, it is possible to produce various functional fermented soy products using dairy propionibacteria.

Even though fruit and vegetable juices seem promising substrates for probiotic bacteria, there are some concerns regarding the viability of probiotics and the possible negative impact of probiotics on the sensory features of the final product. Likewise, several successful strategies have been proposed, in order to tackle these drawbacks, including immobilization/encapsulation. In this manner, the evaluation of immobilization of probiotic *Limosilactobacillus reuteri* DSM 20016, *Bifidobacterium animalis* subsp. *lactis* DSM 10140, and *Lactiplantibacillus plantarum* c16 and c19 in apple-based carriers are discussed by Campaniello et al. Even though, the cells retained their viability during cold storage, a worsening of color with an increase of browning index was observed. The use of alginate and gelatin-coating frustrated the negative effects of both minimally processed fruits (perishable products) and probiotics (browning of apple chips) and assured the retention of sensory scores for a longer time. Coated chips showed higher sensory scores and lower browning index. In addition, gelatin showed better performances in terms of viability, because at 8°C, a significant viability loss of *B. animalis* DSM 10140 was found on alginate-coated chips. The outcome showed that Gelatin-coated apple pieces with *B. animalis* subsp. *lactis* DSM 10140 could be an attractive functional food for a wide audience.

Kimchi is one of the important traditional fermented foods consumed in Korea and other East Asian countries, like Japan and China. Functional beverage production based on kimchi fermentation with an anti-inflammatory probiotic *Limosilactobacillus reuteri* EFEL6901, was discussed by Seon et al. Initially, EFEL6901 cells proved their anti-inflammatory properties through *in vitro* and *in vivo* assessments. Specifically, EFEL6901 cells adhered well to colonic epithelial cells and decreased nitric oxide production in lipopolysaccharide-induced macrophages, while oral administration of EFEL6901 to DSS-induced colitis mice models significantly alleviated the observed colitis symptoms, prevented body weight loss, lowered the disease activity index score, and prevented colon length shortening. These outcomes witnessed the probiotic role of EFEL6901, since prevention of overproduction of pro-inflammatory cytokines, improvements of gut barrier function, and up-regulation of the concentrations of short-chain fatty acids were proved. Finally, *Limosilactobacillus reuteri* EFEL6901was proved as appropriate starter culture in kimchi fermentation with very high growth rates, leading to a probiotic beverage.

New strategies of valorizing fruit and vegetable processing by-products, through the design of new bioprocesses, is undergoing an upsurge in interest. Applications of industrial fermentation to improve nutritional value, or to produce biologically active compounds, is been developing lately. In this sense, the fermentation of a wide variety of by-products including rice, barley, soya, citrus, and milling by-products has been reported and discussed in a minireview paper by Sabater et al. Specifically, various applications have been focused on increasing the nutritional value of vegetable by-products, through the application of a wide variety of microorganisms, such as lactic acid bacteria, *Penicillium, Aspergillus, Yarrowia*, and *Trichoderma* species. Likewise, the main outcomes are the utilization of vegetable by-products as fermentative substrates: (i) for the growth of various microorganisms and (ii) for the production of high biological value protein extracts and a wide variety of functional carbohydrates and glycosidases.

## Author Contributions

The author confirms being the sole contributor of this work and has approved it for publication.

## Conflict of Interest

The author declares that the research was conducted in the absence of any commercial or financial relationships that could be construed as a potential conflict of interest.

## Publisher's Note

All claims expressed in this article are solely those of the authors and do not necessarily represent those of their affiliated organizations, or those of the publisher, the editors and the reviewers. Any product that may be evaluated in this article, or claim that may be made by its manufacturer, is not guaranteed or endorsed by the publisher.
